# Efficient selection and evaluation of transgenic lines of *Crambe abyssinica*

**DOI:** 10.3389/fpls.2013.00162

**Published:** 2013-05-27

**Authors:** Xueyuan Li, Jing Fan, Jens Gruber, Rui Guan, Margrit Frentzen, Li-Hua Zhu

**Affiliations:** ^1^Department of Plant Breeding, Swedish University of Agricultural SciencesAlnarp, Sweden; ^2^Department of Biology, Institute of Biology I, Botany, RWTH Aachen UniversityAachen, Germany

**Keywords:** *Crambe abyssinica*, genetic transformation, hygromycin selection, qRT-PCR, Southern blotting

## Abstract

*Crambe abyssinica* is a dedicated oilseed crop suitable for production of industrial feedstocks. Genetic modification of crambe has progressed substantially in the last few years, but the transformation efficiency needs to be further improved. Meanwhile, developing a reliable molecular system including Southern blot and qRT-PCR analyses is desired for effectively evaluating transgenic lines and gene expression levels of both endogenous and transgenes. In this study, we have developed an efficient transformation protocol with hygromycin as the selective agent for crambe transformation. In the regeneration test, addition of hygromycin at concentration of 5 mg L^−1^ resulted in 18% of shoot regeneration using crambe hypocotyls as explants, while no regeneration occurred when the hygromycin concentration reached 10 mg L^−1^. Based on this result, the hygromycin concentration up to 10 mg L^−1^ was used in the subsequent transformations. The results showed that the transformation efficiency under constant low selection pressure (H3-H3) was similar to that under higher selection pressure first, followed by transfer to lower selection pressure (H10-H3). The PCR, Southern blot and fatty acid composition analyses confirmed the integration of transgenes in the crambe genome. We have also optimized the Southern and qRT-PCR methods for future studies on crambe or related species. For Southern blot analysis on crambe, more than 50 μg DNA is required for a clear band. The choice of enzymes for DNA digestion was not rigid for confirmation of the T-DNA integration, while for determining the copy number of transgenes, suitable enzymes should be chosen. Increasing the enzyme concentration could improve the digestion and 20 μl enzyme was recomended for a complete digestion of up to 80 μg crambe DNA. For qRT-PCR analysis, around 20 days after flowering was observed to be the suitable sampling time for expresseion analysis of genes invovled in the seed oil biosynthesis.

## Introduction

*Crambe abyssinica* is a dedicated oilseed crop for producing industrial feedstocks as it does not outcross with any food oil crops that are currently in commercial production, and thus eliminating the problem of gene flow (Li et al., 2012). The seed oil of crambe is non-edible, which could prevent it entering into the food and feed chain, and thus avoiding the problem of food safety. Meanwhile, the seed oil of crambe contains 55–60% erucic acid, which has a special value for the chemical industry. Moreover, crambe has also a high yield potential and some good agronomic properties, such as potential for phytoremediation of heavy metal contaminated soils and sediments (Chhikara et al., 2012) and tolerance to abiotic stresses such as reduction of soil nematodes, which make it easily accepted by the commercial production after improvement. Owing to these good characters, crambe is an ideal oilseed crop for being a bio-platform for industrial feedstock production.

Genetic modification of crambe has not been possible until very recently. Li et al. (2010, 2011) firstly reported a regeneration protocol with over 95% of regeneration frequency and a transformation protocol with 2.1% of transformation frequency using hypocotyls as explants and kanamycin as selective agent. Using this transformation protocol, Li et al. (2012) successfully transformed three target genes into crambe for increasing erucic acid levels in the seed oil and obtained a transgenic line with 73% of erucic acid content in average compared with 60% in the wild type. Chhikara et al. (2012) reported another protocol with 50–70% regeneration and 6.7–8.3% transformation frequency using also hypocotyls as explants but hygromycin as selective agent. These achievements have demonstrated the feasibility of developing crambe into a bio-platform for industrial feedstock production. However, the information about crambe transformation is still limited. Further improvement of transformation efficiency and optimization of molecular methods for efficient evaluation of transgenic lines are highly desirable.

Hygromycin is the second important selection agent after kanamycin among all antibiotic selection agents suitable for plant transformation (Olhoft et al., 2003). Similar to kanamycin, the function of hygromycin is to inhibit polypeptide elongation in protein synthesis (Gonzalez et al., 1978). It has been reported that hygromycin is more effective for transgenic cell selection than other selective agents in some species. Studies have showed that hygromycin at low concentration could induce somatic embryogenesis and promote morphogenesis, while high doses of hygromycin often cause injury of explants (Park et al., 1995; Aoshima, 2005). To avoid the negative effect of higher concentrations of hygromycin on shoot regeneration and to increase the selection efficiency, the stepwise selection, i.e., a gradual increase or decrease in hygromycin concentration during the selection period, has been reported in some plant species (Boszoradova et al., 2011; Abou-Alaiwi et al., 2012). However, this method has not been applied in crambe transformation.

Different molecular methods are needed for evaluating transgenic materials and for other purposes. Southern blot analysis is commonly used for detecting the T-DNA integration and copy number of transgenes. It has been shown that it is difficult to achieve a satisfactory result by Southern blot analysis on crambe DNA according to our experience and personal communication with some colleagues working on the EU-ICON project (http://icon.slu.se/ICON/). Many factors, such as, the origin of plant material (*in vitro* or greenhouse), the type and amount of restriction enzymes, the duration of digestion and the amount of DNA, need to be optimized to obtain a satisfactory result on Southern blot. qRT-PCR is another useful method commonly used for evaluating gene expression levels of both transgenes or endogenous genes. For the genes involved in seed oil biosynthesis, transcript levels are expected to vary depending on seed development stage. However, such information is not available in crambe. Therefore, it is desirable to improve Southern blot analysis for efficient and reliable evaluation of transgenic lines of crambe and qRT-PCR for finding out the best sampling date for gene expression analysis in relation to seed development. This will provide a sound base for developing crambe into a bio-platform for producing industrial feedstocks.

The objectives of this study were (1) to develop a transformation protocol with hygromycin as selection by a stepwise decrease in hygromycin concentration, (2) to improve Southern blot method, and (3) to find out the best sampling date for qRT-PCR analysis of gene expression levels for genes invovled in seed oil biosynthesis.

## Materials and methods

### Plant material

Seeds of *Crambe abyssinica* cv. Galactica, kindly provided by Dr. Eibertus N. van Loo, were used in this study.

### Vector and *Agrobactium* strain for transformation

**Figure 1:**
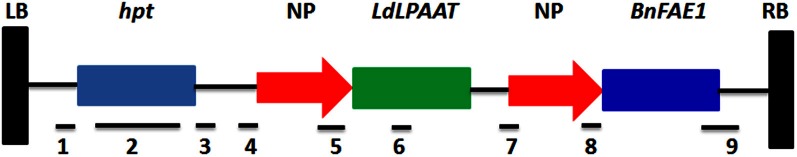
**Schematic map of the vector used in this study.** Horizontal bars under genes or promoters represent the sites of restriction enzymes and the hpt probe used for Southern blot analysis. 1: *Xho*I; 2: hpt probe; 3: *Sma*I, *Xho*I, *Eco*RV; 4: *Eco*RI, *Sma*I, *Bam*HI, *Xba*I, *Sal*I, *Bam*HI, *Eco*RV; 5: *Eco*RV, *Eco*RI; 6: *Bam*HI; 7: *Xba*I, *Eco*RV; 8: *Eco*RV, *Eco*RI, *Hin*dIII; 9: *Hin*dIII, *Sal*I, *Pst*I. LB, left border; *hpt*, hygromycin gene; NP, napin promoter; RB, right border.

The *Agrobacterium tumefaciens* strain AGL-1 harboring the binary vector pCAMBIA, the same as pHan-vector according to Li et al. (2012) was used for transformation. The vector harbors the *hpt* (hygromycin phosphotransferase) gene for plant selection and also the *BnFAE1* and *LdLPAAT* genes for increasing the erucic acid content. The *BnFAE1* gene is responsible for the carbon chain elongation during the fatty acid synthesis, while the *LdLPAAT* gene is responsible to add erucic acid into the second position (*sn*-2) of the glycerol backbone (Li et al., 2012). Both *BnFAE1* and *LdLPAAT* genes are under the seed specific napin promoter, while *BnFAE1* is under the *FAE1* terminator and *LdLPAAT* under the napin terminator. The *hpt* gene is under 35S-promoter. The schematic map of the vector is presented in Figure 1.

### *In vitro* culture and biotron conditions

All *in vitro* cultures were maintained in a climate chamber which has the temperature of 25/18°C (day/night) under a photoperiod of 16 h at 40 μmol m^−2^ s^−1^ light intensity (cool white fluorescent tubes). The transgenic lines and wild type plants were grown in the biotron at 21/18°C (day/night) under a photoperiod of 16 h at 250 μmol m^2^ s^−1^ light intensity and a humidity of 60%. The plantlets were watered regularly with slow release fertilizer (N:P:K = 21:3:10). The harvested seeds were kept at 4°C before oil composition analysis.

### Methods

#### Seed germination

Seeds without silique were surface-sterilized using 15% calcium hypochlorite (CaCl_2_O_2_) for 25 min and rinsed thoroughly with sterile water. Thirty surface-sterilized seeds were planted on a germination medium in each Petri dish that was placed in a sterile Magenta box. The germination medium contained half-strength MS (Murashige and Skoog, 1962), 10 g L^−1^ sucrose, 7 g L^−1^ Bacto agar at pH 5.8. The boxes were placed in the climate chamber in dark for 3 days.

#### Hygromycin tolerance test

The test was conducted before transformation using hypocotyls as explants. The cotyledons and shoot apical meristems of 3 days old seedlings were removed and hypocotyls of about 1 cm from the biological top were cut into 2–3 mm in length. The explants were cultured on the regeneration medium (RM) containing MS, 30 g L^−1^ sucrose, 10 μM thidiazuron (TDZ), 2.7 μM alpha-naphthalene acetic acid (NAA), 0.5 g L^−1^ MES, and Gelrite 2.5 g L^−1^ at pH 5.8, supplemented with hygromycin at concentrations of 0, 5, 10, and 20 mg L^−1^. For each concentration, 100 hypocotyls were used. The experiment was repeated twice. The explants were transferred to fresh medium every 2–3 weeks and the results were recorded after 2 months.

#### Plant transformation

The bacteria were cultured in liquid LB medium with appropriate antibiotics for about 18 h. After centrifugation at 4000 rpm for 15 min, the pellet was suspended in liquid MS20 medium (full MS, 20 g L^−1^ sucrose, pH 5.2, 200 μM acetosyringone) to a concentration around 0.4 at OD_600_. The hypocotyl explants as stated above were pre-cultured on filter paper for 3 days on the RM medium prior to *Agrobacterium* infection. The pre-cultured explants were thoroughly washed in the bacterial suspension for 1–2 min, dry blotted in a sterile filter paper and co-cultured on the co-culture medium with filter paper for 3 days in light. The co-culture medium was the same as the RM medium, but contained 200 μM acetosyringone. After co-culture, the explants were washed with water containing 200 mg L^−1^ cefotaxime, dry blotted on sterile filter paper and placed on the selection medium, which consisted of RM, 0.5 mg L^−1^ AgNO_3_, 100 mg L^−1^ cefotaxime, 100 mg L^−1^ ticarcillin, and different concentrations of hygromycin depending on experiment (see Table 3 for the detail). The explants were transferred to fresh selection medium every 2 weeks. The concentrations of hygromycin were changed after 2 weeks.

#### Verification of transgenic lines

Both PCR and Southern blot analyses were carried out to confirm the transformation events. The total genomic DNA was extracted from *in vitro* grown shoots using the CTAB method as described by Aldrich and Cullis (1993). For PCR analysis, the *hpt* gene was amplified to verify the integration of the transgene. The primers used were: forward 5′-GATGTTGGCGACCTCGTATT-3′ and reverse 5′-GATGTAGGAGGGCGTGGATA-3′, yielding a 579 bp product. The PCR analysis was performed according to Zhu and Welander (2000). For Southern blot hybridization, the amount of genomic DNA, the type and amounts of restriction enzymes as well as the duration of digestion were tested. The enzymes were the products from Fermentas (St. Leon-Rot, Germany) and digestion was performed according to the manufacturer's instruction. Southern blot hybridization was based on the non-radioactive DIG system from Roche (van Miltenburg et al., 1995). The hpt probe was synthesized according to Zhu et al. (2001, 2008). For comparison of DNA amounts used for obtaining clear bands using nptII probe, DNA from one transgenic crambe line with a single copy of *nptII* gene, previously reported by Li et al. (2012) and DNA from one Arabidopsis transgenic line with *npt* gene, obtained from another study, were used. The nptII probe was described by Zhu et al. (2008).

#### Oil analysis

For extraction of the seed oil, i.e., triacylglycerols, a single seed sample was grounded in a mortar with 500 μl hexane, followed by an addition of 500 μl hexane for complete homogenization of the tissues. The extraction solution was passed through a Pasteur pipette with a glass wool plug to remove seed residues and then dried under nitrogen stream; thereafter 2 ml methylation solution (2% H_2_SO_4_ in water-free methanol) was immediately added and methylated at 95°C for 45 min. After methylation, 0.5 ml hexane and 2 ml water were added. After brief vortexing and centrifugation at 350 g for 3 min, the hexane phase containing FA methyl esters (FAMEs) was transferred into a GC vial for FA composition analysis using Shimadzu GC-17A gas chromatograph with WCOT Fused Silica CP-Wax 58 with a FID detector (Shimadzu Corporation, Kyoto, Japan). Peaks were identified according to their retention time in comparison with a standard FAME mixture, and results are expressed as area percentage of each FAME in all detectable peak areas.

#### Sampling time for gene expression analysis

To determine a proper sampling time for evaluating transcript levels of genes involved in the seed oil biosynthesis, we have investigated the expression of endogenous genes *CaFAE1* and *CaFAD2* at the different stages of seed development. We did not measure the gene expression levels of transgenes *BaFAE1 and LdLPAAT* because the seeds from T1 generation are heterogeneous, which will make qRT-PCR results difficult to interpret. The developing seeds were collected every 3 days from day 10 after flowering to day 40 when the seeds were fully matured. The selected seeds were frozen in the liquid nitrogen and stored at −80°C for further use.

#### RNA extraction

Total RNA was extracted from immature seeds collected at different days (see Figure 8 for detail) after flowering using the RNeasy Plant Mini Kit (Qiagen, Hilden, Germany) following the manufacturer's instructions. A single seed was used as one replicate with three biological samples and four technical replicates for each qRT-PCR. Residual genomic DNA was removed through DNase treatment using TURBO DNA-free (Ambion, Austin, TX).

#### qRT-PCR analysis

First-strand cDNA was synthesized from 400 ng of total RNA in 20 μL with RevertAid H Minus First Strand cDNA Synthesis Kit (Fermentas, St. Leon-Rot, Germany). The cDNA was diluted four times and 2 μL was used for each 20 μL qRT-PCR analysis using Applied Biosystems 7300 Real-Time PCR System (Applied Biosystems, Foster City, CA) with Platinum SYBR Green qPCR SuperMix-UDG module (Invitrogen, Carlsbad, CA). The PCR programme was 50°C for 2 min and 95°C for 2 min, followed by 40 cycles of 95°C for 15 s and 60°C for 30 s. Melting curve analysis was performed to confirm the product specificity. The primers for *CaFAE1* and *CaFAD2* were selected from several sets of primers tested in the preliminary analysis. Gene expression was normalized to the reference gene *AtUBC21* (Table 1), which has been proved to be stably expressed during different developmental stages in Arabidopsis (Czechowski et al., 2005). The partial sequence of crambe ubiquitin gene (unpublished) has shown that the primers used for qRT-PCR are 100% homologous between Arabidopsis and crambe.

**Table 1:** **The sequences of the primers used for qRT-PCR**.

**Gene**	**Forward primer (5′ – 3′)**	**Reverse prime (5′ – 3′)**
*AtUBC21*	TGCGACTCAGGGAATCTTCT	TCATCCTTTCTTAGGCATAGCG
*CaFAD2*	CCGTGAACGTCTCCAGATAT	CGTTGACTATCAGAAGCCGA
*CaFAE1*	CCTCCCCGGAAGACTTTTG	CATGCTTGAGTTCACCACAAG

## Results and discussion

### Hygromycin tolerance test

**Figure 2:**
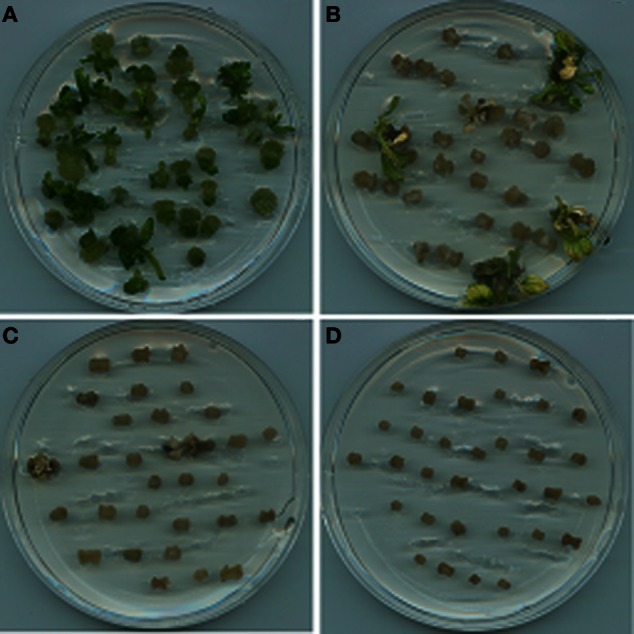
**Results of shoot regeneration from hypocotyl explants grown on the regeneration medium supplemented with hygromycin at concentrations of 0 mg L^−1^ (A), 5 mg L^−1^ (B), 10 mg L^−1^ (C), and 20 mg L^−1^ (D)**.

A suitable selectable pressure is important for achieving an efficient transformation frequency. If the selection pressure is too high, no transgenic lines will be recovered, while more escapes will be produced if the selection pressure is too low. A wide range of hygromycin concentrations have been used for transformation of various plant species, ranging from 3 mg L^−1^ for rapeseed (Liu et al., 2011; Zhang et al., 2011) to 100 mg L^−1^ for onion (Eady and Lister, 1998). In this study, hygromycin at 5 mg L^−1^ could already significantly reduce the regeneration frequency (18%) of crambe hypocotyls compared to 94% when no hygromycin was added in the medium. The growth of regenerants was also obviously retarded on the medium with 5 mg L^−1^. Although about 2–3% regeneration could occur when the hygromycin concentration reached 10 mg L^−1^, the regenerants could only survive for a very short time, while no regeneration occurred when hygromycin reached 20 mg L^−1^ (Table 2, Figure 2). Our result is not in line with that reported by Chhikara et al. (2012) where 20 mg L^−1^ hygromycin was used for crambe transformation. This discrepancy might be due to the different genotypes used in both studies. It has been reported that the concentrations of hygromycin used for transformation vary greatly depending on genotype, type of explants and even explant size (Milojević et al., 2012). Since 10 mg L^−1^ hygromycin was proved to be lethal to the wild type crambe, we thus used this concentration as the highest limit in the subsequent transformation experiments.

**Table 2:** **Regeneration results of the hygromycin test on crambe hypocotyls**.

**Hygromycin concentration (mg L^−1^)**	**Explant No.**	Regeneration frequency (%)^*^
0	100	94 a
5	100	18 b
10	100	0 c
20	100	0 c

* Results were the means of two experiments; only shoots survived for more than 1 month were taken into account for statistical analysis. Different letters indicate significant differences at P = 0.05. Data were analysed with Duncan's multiple test using the Statgraphics program.

### Tranformation efficiency

As stated earlier, the hygromycin concentration used for genetic transformation depends largely on plant species. Some species are extremely sensitive to hygromycin and low concentrations of hygromycin should be used to ensure an efficient selection, while others are rather tolerant to hygromycin and much higher doses are required to eliminate the escapes. It has been reported that higher hygromycin concentrations would cause interference of regeneration process, and thus inhibiting the growth of transgenic shoots (Horn et al., 1988; Humara and Ordas, 1999). Furthermore, cultures under high selection pressure may produce a high proportion of transgenic lines with multiple copies of transgenes (Hamill et al., 1987; Dekeyser et al., 1989; Dalton et al., 1995). To overcome this problem, delayed selection or stepwise selection methods have been studied in some plant species. The delayed selection means delaying transfer of explants to selection media after *Agrobacterium* co-culture, while the stepwise selection means that applying a lower concentration of selective agent first, followed by a gradual increase in concentration to a certain level (Visser et al., 1989; Bhuiyan et al., 2011; Milojević et al., 2012). It has been supposed that the initially low selective pressure allows the recovery of transformed cells, while the high selective pressure in subsequent transfers will eliminate untransformed cells and allow only the transformants to grow. The main concern of this method is that the transformed cells may not initially be able to compete with the untransformed cells under a low selection pressure; as a result, the growth of transgenic cells would be retarded or even eliminated.

In this study, we have evaluated an opposite selection method, that is, using high selection pressure for a short period to eliminate untransformed cells first, followed by a decrease in the selection pressure allowing the transformed cells to recover. Among the hygromycin concentrations tested as selective agent, the treatments H3 to H3, H5 to H3, H5 to H5 and H10 to H3 were equally efficient in term of percentage of putative transgenic lines and escape production as well as transformation frequency. Treatment H10 to H5 guaranteed from escape production but provided a lower percentage of transgenic lines compared to H3 to H3, H5 to H3, H10 to H3, and a lower of transformation efficiency in comparison to H10 to H3. From these results, we can conclude that crambe is indeed very sensitive to hygromycin and the regeneration occurred better under constant low selection pressure or higher selection pressure followed by transfer to low selection pressure (Table 3). From economic and practical point of view, it might be better to maintain the cultures under low selection pressure all the time.

**Table 3:** **Transformation results of crambe using hypocotyls as explants and hygromycin (H) as selective agent**.

**Treatment^1^**	**Total no. of explants used**	**Putative transgenic lines (%)^3^**	**Escapes (%)**	**Transformation frequency (%)^3^**
H3 → H3	510	9.8 a	1.8 a	8.0 ab
H5 → H3	483	9.3 a	1.4 ab	7.9 ab
H5 → H5	499	8.0 ab	0.8 ab	7.2 ab
H10 → H3	525	10.7 a	0.8 ab	9.9 a
H10 → H5	296	4.7 b	0 b	4.7 b

1 Figures after H represent hygromycin concentration in mg L^−1^ in the regeneration medium. Arrow represents transfer of cultures from one concentration to another. The transfer to a new hygromycin concentration occurred after the first transfer and the concentration was maintained in the subsequent transfers.

2 Putative shoots survived on the shoot proliferation medium with hygromycin at 10 mg L^−1^ less than 2 months. Putative transgenic % was calculated by dividing the number of putative transgenic lines by the total explant number used for transformation.

3 Transformation efficiency % was calculated by dividing the number of transgenic lines by the total explant number used for transformation.

Different letters in each column indicate significant differences at P = 0.05. Data were analysed with Duncan's multiple test using the Statgraphics program.

### Fatty acid composition in the seed oil

Twenty transgenic lines with *LdLPAAT* and *BnFAE1* genes were planted in the biotron for producing T1 seeds. The seeds from 15 lines showed the altered fatty acid composition in the seed oil. Since the lines are still heterozygous and great variation exists among the individuals within the same line, we here only present some single seeds with the extreme values to confirm the integration and expression of the transgenes in Table 4. The erucic acid (22:1) content was clearly increased in the best single seeds, but not as high as that reported earlier with the same construct with *nptII* as selectable gene (Li et al., 2012). It should be noticed that the oleic acid level (18:1) in some transgenic seeds was drastically increased, up to 56.2% compared to 14.9% for the wild type, suggesting a strong co-suppression of the introduced and endogenous *FAE1* genes. This is in accordance with the previous report by Li et al. (2012).

**Table 4:** **Fatty acid composition (% based on peak area) in the seed oil of single T1 seeds with either higher 22:1 or 18:1 in transgenic seeds (H) with the *LdLPAAT* and *BnFAE1* genes in comparison with the wild type (WT)**.

**Seed**	**18:1**	**18:2**	**18:3**	**20:1**	**22:1**	**24:1**
WT^1^	14.9	7.4	6.1	2.9	58.4	1.2
H1-1	7.1	6.6	8.4	4.3	63.1	1.3
H1-2	9.7	6.1	9.1	3.0	62.1	1.3
H3-7	8.9	5.6	6.1	4.9	65.2	1.6
H11-15	8.3	6.9	8.3	4.3	62.3	1.2
H20-28	9.7	7.5	7.0	3.9	62.6	1.4
H6-25	51.2	11.9	9.9	6.6	10.0	0.5
H9-5	49.0	9.3	5.8	8.9	16.5	0.4
H19-21	49.7	13.2	9.1	5.6	12.9	0.4
H19-24	54.2	11.6	8.2	4.7	11.8	0.4
H19-25	56.2	14.0	9.5	5.1	5.7	0.2

1 Average value of 10 seeds.

### PCR and southern blot analysis

**Figure 3:**
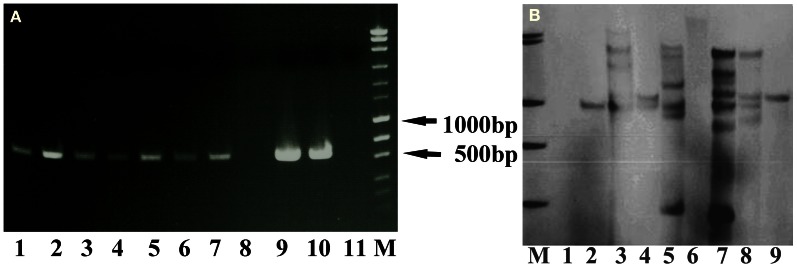
**PCR and Southern blot results of the putative transgenic lines of crambe. (A)** The PCR product size of the *hpt* gene is 579 bp. Lane 1–10, putative transgenic lines. Lane 11, wild type. M, molecular markers (MassRuler “Express DNA ladder Mix” from Fermentas). **(B)** Southern blot analysis of genomic DNA digested with *Bgl*II and hybridized with the hpt probe. M, DIG labeled molecular markers. Lane 1, wild type. Lane 2–9, transgenic lines.

The PCR results showed the presence of the *hpt* gene except for lane 8 (Figure 3A), while the Southern blot result confirmed the stable integration of the *hpt* gene as clear bands were visible after being hybridized with the hpt probe (Figure 3B). The band patterns suggest that the copy number of the *hpt* gene ranged from 1 to at least 6.

### Efficient evaluation of transgenic lines with southern blot analysis

**Figure 4:**
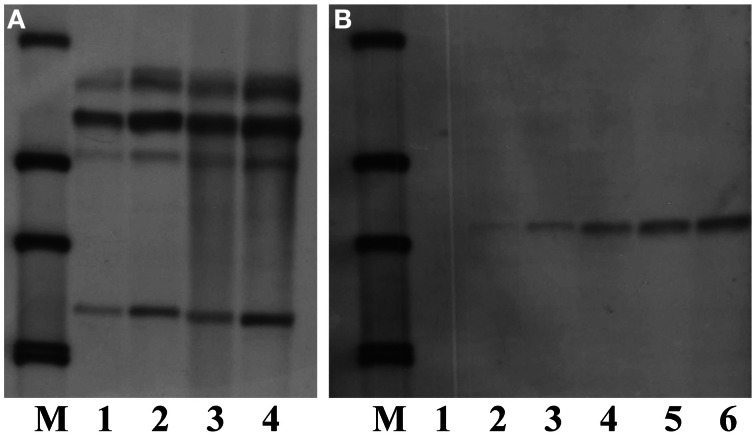
**Southern blot analysis showing different amounts of DNA used for digestion with *Hin*dIII and hybridized with the nptII probe according to Li et al. (2012). (A)** DNA from transgenic Arabidopsis with the *nptII* gene, lane 1, 5 μg; lane 2, 10 μg; lane 3, 20 μg; lane 4, 50 μg. **(B)** DNA from transgenic crambe with the *nptII* gene, lane 1, 5 μg; lane 2, 10 μg; lane 3, 20 μg; lane 4, 50 μg; lane5, 80 μg; lane 6, 100 μg. M, DIG labeled molecular markers.

Sucessful performance of Southern blot analysis relies on good quality and quantity of genomic DNA, a correct choice of restriction enzymes and suficient amounts of restriction enzymes as well as an appropriate duration of DNA digestion. To optimize the conditions for Southern blot analysis for cambe, a series of experiments were carried out. The results showed that, compared with Arabidopsis, a large quantity of DNA was needed for Southern blot analysis of crambe, for example, 5 μg DNA were suficient to yield clear bands for Arabidopsis, while 50–80 μg DNA were required for producing strong bands for crambe (Figure 4).

**Figure 5:**
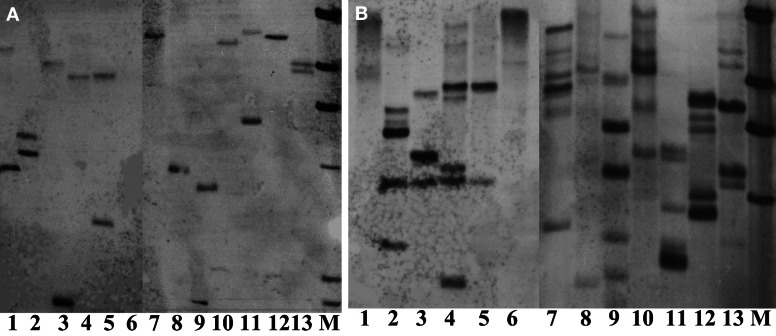
**Southern blot analysis on DNAs from two transgenic lines with 2 (A) and multiple copies (B) of transgenes respectively, digested with 13 different restriction enzymes and hybridized with the hpt probe. (A,B)** From lane 1 to 13: *Bgl*II, *Kpn*I, *Sac*I, *Sal*I, *Sma*I, *Stu*I, *Hin*dIII, *Xho*I, *Xba*I, *Pst*I, *Eco*RV, *Dra*I, and *Bam*HI. M, DIG labeled molecular markers. DNA, 80 μg. Enzyme, 20 μl.

Thirteen restriction enzymes were arbitrarily chosen for their suitability in digesting the genomic DNA of crambe for Southern blot analysis. The principle to choose these enzymes was that they do not cut the *hpt* gene. The results showed that nearly all enzymes tested could produce band(s) using transgenic crambe lines with ether two or multiple copies of the transgene (Figure 5), suggesting that most commonly used restriction enzymes could be used for crambe DNA digestion for Southern blot analysis to confirm the T-DNA integration. However, not all of them are siuitable for determining the copy number of transgenes. *Bam*HI, *Dra*I, *Eco*R5, *Hin*dIII, *Kpn*I, *Pst*I, and *Xba*I gave 2 bands with the DNA from one transgenic line (Figure 5A) and resulted in 6–7 bands with the DNA from another transgenic line (Figure 5B). For single or double copies, the abovementioned enzymes appear to be suitable for determining the copy number, while for multiple copies, *Bam*HI, *Hin*dIII, *Kpn*I, and *Xba*I appear to give a relatively better band separation. In general it is difficult to get clear and better band separation when the copy number of transgenes is high than two.

**Figure 6:**
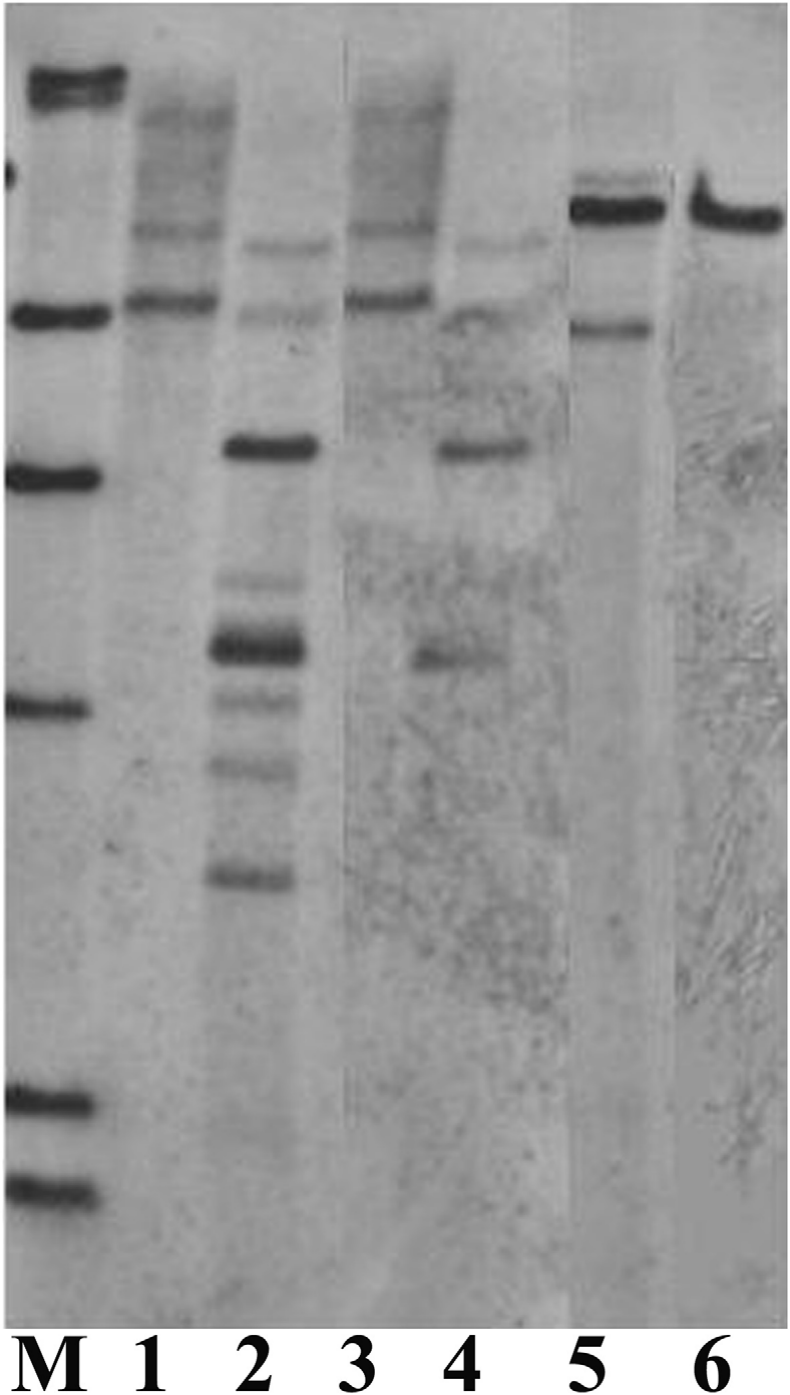
**Southern blot analysis on DNAs from 3 different transgenic lines (one transgenic line for one enzyme), digested with different amounts of the enzymes.** M, DIG labeled molecular markers. Lane 1: *Pst*I, 20 μl; Lane 2: *Kpn*I, 20 μl; Lane 3: *Pst*I, 10 μl; Lane 4: *Kpn*I, 10 μl; Lane 5: *Bgl*II, 20 μl; Lane 6: *Bgl*II, 10 μl. DNA, 80 μg.

The analysis on the amount of restriction enzymes showed that it appears to be enzyme-dependent, for example, there was no clear difference between 10 and 20 μl for *Pst*I, but 20 μl gave more and clearer bands than 10 μl for *Kpn*I and *Bgl*II (Figure 6). The study on the enzyme digestion duration showed no obvious differences among the digestion durations of 1, 10, and 20 h (data not shown). This might be due to the fast digestion enzymes used in this study which do not normally require longer digestion. However, overtime digestion did not give any negative effect on Southern blot result.

### Gene expression levels during the seed development

**Figure 7:**
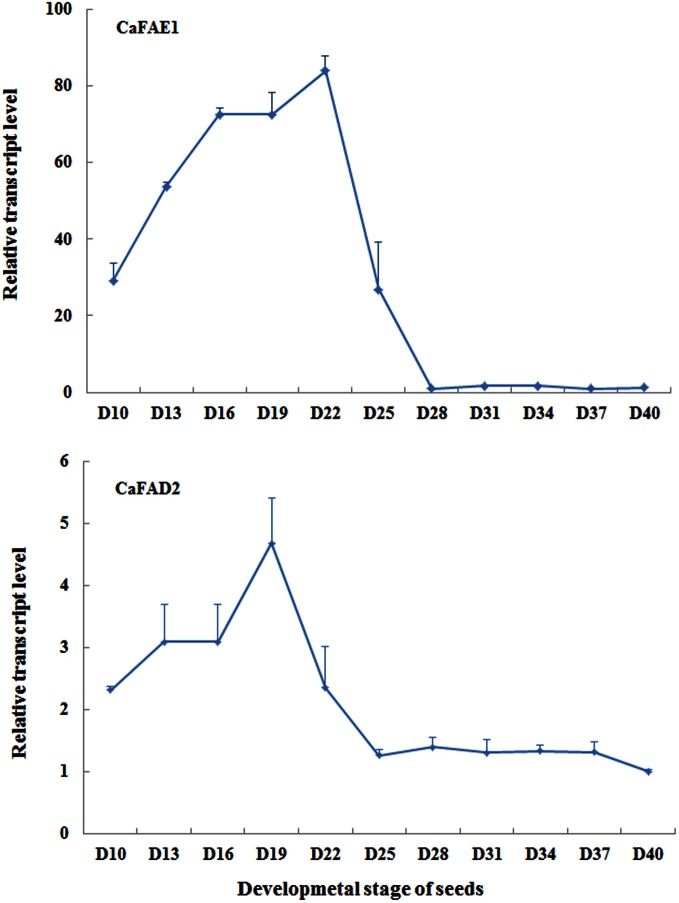
**The qRT-PCR results showing the relative expression levels of *CaFAE1* And *CaFAD2* mRNA during the seed development.** D, days after flowering. Data is the average of three biological replicates for each time point.

**Figure 8:**
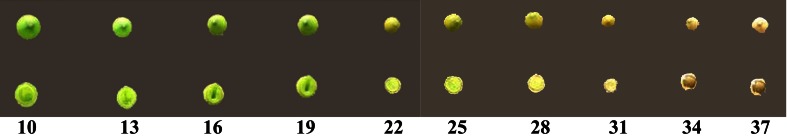
**Crambe seeds at different development stages.** The upper panel is the whole seed and lower panel is the cross section of the seeds, but the whole seed with half silique removed at the last two stages. Figures represent days after flowering.

For detecting expression levels of genes associated with seed oil biosynthesis using qRT-PCR, an appropriate seed sampling date is important because the expression level of target genes can vary depending on seed developmental stage. This principle applies to both endogenous genes and transgenes with seed specific promoter. It should be also pointed out that the speed of seed development relies largely on growth conditions, for instance, low temperature and light could delay the seed development. Under our growth conditions, around 20 days after flowering is the best time for sampling when evaluating expression levels of *CaFAD2* and *CaFAE1* genes (Figure 7), which encode the important enzymes involved in the seed oil biosynthesis. According to our observation, this is the time when the seed coat begins to become hard, but still green (Figure 8).

### Conflict of interest statement

The authors declare that the research was conducted in the absence of any commercial or financial relationships that could be construed as a potential conflict of interest.
